# Psychosocial Stress, Cortisol Levels, and Maintenance of Vaginal Health

**DOI:** 10.3389/fendo.2018.00568

**Published:** 2018-09-24

**Authors:** Emmanuel Amabebe, Dilly O. C. Anumba

**Affiliations:** Academic Unit of Reproductive and Developmental Medicine, University of Sheffield, Sheffield, United Kingdom

**Keywords:** stress, corticotropin-releasing hormone, cortisol, estrogen, vaginal microbiome, vaginal microbiota

## Abstract

Stress stimuli are ubiquitous and women do not enjoy any exemptions. The physiologic “fight-or-flight” response may be deleterious to the female lower genital tract microbiome if the stress stimuli persist for longer than necessary. Persistent exposure to psychosocial stress and stimulation of the hypothalamic-pituitary-adrenal (HPA) and sympathetic-adrenal-medullary (SAM) axes, and associated hormones are risk factors for several infections including genitourinary tract infections. Though this could be due to a dysregulated immune response, a cortisol-induced inhibition of vaginal glycogen deposition may be involved especially in the instance of vaginal infection. The estrogen-related increased vaginal glycogen and epithelial maturation are required for the maintenance of a healthy vaginal ecosystem (eubiosis). The ability of cortisol to disrupt this process as indicated in animal models is important in the pathogenesis of vaginal dysbiosis and the subsequent development of infection and inflammation. This phenomenon may be more crucial in pregnancy where a healthy Lactobacillus-dominated vaginal microbiota is sacrosanct, and there is local production of more corticotropin-releasing hormone (CRH) from the decidua, fetal membranes and placenta. To highlight the relationship between the stress hormone cortisol and the vaginal microbiomial architecture and function, the potential role of cortisol in the maintenance of vaginal health is examined.

## Introduction

Stress is an organism's attempt to adequately respond to either internal or external threats or injuries. It refers to any physical or psychological challenge that threatens or has the potential of threatening the equilibrium of an organism's internal milieu (homeostasis) (1–3). Such challenges could be life events, situations, emotive feelings, and interactions that adversely affect the individual's wellbeing or trigger perceived harmful responses. Psychosocial stress stimuli comprise life experiences that include changes in personal life and relationships, occupation, housing, family composition, and domestic violence necessitating adaptive survival behaviors/responses from the affected individual (4, 5). Stress stimulates the hypothalamic-pituitary-adrenal (HPA) axis, which eventually leads to elevated levels of cortisol. As a downstream effector of the stress-induced neuroendocrine response, cortisol exerts global effects in the body to maintain homeostasis and enhance the organism's capacity to respond to and grapple with physical and emotional stresses (6). For instance, it primes the organism for “fight or flight” by promoting energy metabolism via glycogenolysis, gluconeogenesis, proteolysis and lipolysis, as well as regulates several immune and inflammatory responses (7). Furthermore, it increases blood pressure, has diverse bone effects, elicits both positive and negative effects on cell growth, and facilitates apoptosis in certain cell types, including certain neuronal cells (8). Normal immune function may be impaired or dysregulated by exposure to chronic stress through the HPA axis and the sympathetic-adrenal-medullary (SAM) axis, resulting in the chronic production of glucocorticoid hormones and catecholamines (9)

Cortisol is a steroid (glucocorticoid) hormone produced by the zona fasciculata of the adrenal cortex within the adrenal gland. It is released by the adrenal glands as part of the fight-or-flight mechanism in response to stress or fear, but has been described as public health's foremost enemy (10). Its functions include the modulation of increased blood sugar through gluconeogenesis and induction of insulin resistance (11, 12), metabolism of fat, protein, and carbohydrates, growth and reproduction, immune suppression (13), sodium-potassium transport (14, 15), cognition and memory (13, 16), and the regulation of bone formation (17).

The release of cortisol is regulated by the HPA axis via the secretion of corticotropin-releasing hormone (CRH) by the paraventricular nucleus (PVN) of the hypothalamus, which stimulates corticotrophs in the anterior pituitary to secrete adrenocorticotropic hormone (ACTH, corticotropin), which travels through the bloodstream to the adrenal cortex. ACTH stimulates the synthesis of cortisol and other glucocorticoids. Cortisol ultimately inhibits the HPA neuroendocrine response via a negative feedback mechanism to restore a steady state (13, 18). Cortisol has a circadian rhythm with lowest levels at midnight and peak levels (~399 nmol/l) in the morning (around 8:30) (19).

Hitherto, most studies have attributed the pathogenesis of stress-related vaginal dysbiosis solely to impaired immune function and loss of *Lactobacillus* species dominance (9, 20), while others have merely enumerated the use of corticosteroids as a factor associated with Bacterial vaginosis (BV). However, the mechanisms by which cortisol, the classical stress hormone, modulates the estrogen-induced deposition and accumulation of glycogen in the vaginal epithelium and the implications for maintenance of vaginal homeostasis has received little attention. Due to the importance of the association between vaginal glycogen, *Lactobacillus* species dominance and low pH for the reproductive health of women, i.e., reducing the risk of sexually transmitted infections (STIs), BV, and preterm labor (21), this review examines the vaginal glycogen response induced by estrogen and the potential repressive role of cortisol.

### Literature search

With the use of words and phrases including (but not limited to) “stress and vaginal health,” “stress and bacterial vaginosis,” “stress and vaginal infection,” “stress and immune function,” “stress and vaginal *Lactobacilli*,” “stress, infection and preterm birth,” “corticosteroids, cortisol and bacterial vaginosis,” “cortisol and vaginal infection,” “cortisol and vaginal *Lactobacilli*,” “cortisol and vaginal glycogen,” “estrogen, vaginal glycogen and *Lactobacillus dominance*,” a comprehensive search was conducted for scientific, peer-reviewed and published original research articles involving both humans and animal models, as well as review articles in PubMed/MEDLINE and Web of Science databases between December 2017 and August 2018. In order to comprehensively review relevant literature relating to psychosocial stress, cortisol levels, and maintenance of vaginal health, publication dates were not restricted. Articles not written in English or including other hormones not directly involved in the HPA or SAM stress axes and vaginal microbial composition (e.g., insulin), were not included.

## Normal vaginal microenvironment

The human vagina is not only a passage for sperm, menstruum, and the baby but also a particularly versatile organ with a protective epithelium and a rich diverse microbial landscape (22). The vaginal microbiome comprises of a stratified squamous non-keratinized epithelium overlaid by a mucin-rich mucus layer and provides an attachment surface for the commensal and most dominant lactic acid producing *Lactobacillus* species. The most often identified species of *Lactobacillus* are *L. crispatus, L. jensenii, L. gasseri*, and *L. iners* (23, 24). Other bacteria endogenous to the normal vaginal microenvironment albeit with low virulence capacity include *Gardnerella, Prevotella, Fusobacterium, Atopobium, Streptococcus, Staphylococcus, Peptostreptococcus, Porphyromonas* etc. These potentially pathogenic bacteria are kept dormant by the acidic milieu (pH 3.5–4.5) created by *Lactobacilli* amongst other protective mechanisms including production of lactic acid (~110 mM) (25, 26), hydrogen peroxide (H_2_O_2_), antimicrobial peptides and by competitive exclusion i.e., physically preventing the attachment of pathogens to vaginal epithelium (22, 27, 28). The commensal and potentially harmful vaginal microorganisms, their genes and products collectively form the vaginal microbiota that dwell in a regulated mutualistic relationship with the host vaginal epithelium to form the microbiome (29).

The vaginal microbiota in childhood until puberty is dominated by anaerobes due to low glycogen content, a decrease in *Lactobacilli* and other acid-producing microbes and a more alkaline pH (30). This increases their susceptibility to genital infections (e.g., vulvovaginitis) by a variety of aerobic and anaerobic pathogens including *S. pyrogens, N. gonorrhea, E. coli, E. faecalis, C. vaginalis, Mycoplasmas, Diphtheroids, Bacteroides, S. epidermidis, C. albicans* etc. (31–35). Fortunately, due to lack of exposure to sexual intercourse (coitus), the incidence of genital tract infections is low in children except in cases of child sexual abuse (36–38). At puberty, under the influence of rising estrogen levels, the vaginal epithelium thickens and stratifies, intracellular glycogen levels increase and undergo cyclical changes, cervicovaginal secretions are produced, and proliferation of lactic acid-producing lactobacilli commence (30, 39). The increasing production of lactic acid by *Lactobacillus* suggests there is a fermentable substrate present in the vagina. Glycogen is identified as the suitable carbohydrate substrate as an association between high acid secretion and presence of glycogen in the vagina was demonstrated over 80 years ago (40). Vaginal glycogen is degraded by host α-amylase into maltose, maltotriose and α-dextrins, which are then converted to lactic acid by *Lactobacilli* (21, 39, 41–43). Elevated estrogen and glycogen levels promote increased thickness of the stratified squamous epithelium and protective mucus layer of the vagina (44). Lactic acid at physiological concentration (1% w/v, ~110 mM) (25, 26, 45) reduces the vaginal pH, which encourages the proliferation of *Lactobacilli* and inhibits the growth of the anaerobes and viruses capable of causing infection (26, 39, 45–49). It also exhibits some immunomodulatory effects on cervicovaginal epithelial cells and other cell types. It stimulates an anti-inflammatory state through the increased production of IL-1 receptor antagonist (IL-1RA) from cervicovaginal epithelial cells and inhibits the activation of nuclear factor- κB (NF-κB) in peripheral blood mononuclear cells and monocytes-macrophages (50, 51), which promotes the transcription of pro-inflammatory target genes. In addition, it inhibits the Toll-like receptor (TLR)-induced production of inflammatory mediators from cervicovaginal epithelial cells. Both D- and L-lactic acid exhibit these anti-inflammatory effects that are potentiated by low pH < 3.86 (22, 49, 52). The homeostatic vaginal environment created by lactobacillus-dominant microbiota is temporarily altered during menstruation when there is a decline in estrogen and glycogen levels, and neutralization of the acidic pH promoting the growth of pathogenic bacteria such as *Gardnerella, Prevotella*, and *Atopobium* (53). After menstruation, the normal pH and Lactobacillus dominance are re-established as estrogen levels begin to rise again (54).

The vaginal microbiota in normal pregnancy is predominated by *Lactobacilli* species and is more stable than that in non-pregnant state (55, 56). This can be explained by the higher level of estrogen during pregnancy resulting in increased vaginal glycogen deposition, which enhances the proliferation of lactobacilli-dominated vaginal microbiota. As discussed later in this article, oestriol, which is one of three major endogenous estrogens produced in significantly larger amounts by the placenta during pregnancy (57), was as potent as 17β-oestradiol (the most common form of estrogen in non-pregnant premenopausal women) in stimulating vaginal glycogen deposition (58).

Following menopause, *Lactobacilli* dominance decreases secondary to diminished estrogen levels (59, 60) and cessation or decline of glycogen production (61). Loss of *Lactobacilli* increases vaginal pH to a more alkaline environment, providing a conducive habitat for colonization by anaerobes (possibly of fecal origin) and other pathogens (30, 62). For instance, post-menopausal women showed lower free genital fluid glycogen levels (41), and harbored more of *G. vaginalis, Bacteroides, Peptostreptococcus, Streptococcus*, and *Prevotella* (63) compared to premenopausal women who had higher free glycogen (41) and preponderance of *L. crispatus* and *L. iners* (64). Topical or systemic hormone replacement therapy and probiotics restore vaginal *Lactobacillus* dominance and homeostasis with increased acidity (65), and improves vaginal symptoms in post-menopausal women (44). In some cases, there is a decrease in the prevalence of anaerobic gram-negative rods and vaginal pH while the aerobic isolates including *Lactobacilli* remain fairly constant (65).

Though progesterone alone and in combination with estrogen greatly increases the glycogen concentration in the vaginal epithelium in squirrel monkeys (66), it does not appear to have the same effect in humans (58). For instance, progestin-only contraceptive drugs such as Depomedroxyprogesterone acetate (Depo-Provera) can produce a systemic hypoestrogenic state associated with slight thinning of the glycogen vaginal epithelial layer and reduced *Lactobacillus* colonization compromising the vaginal barrier against infection (67, 68). In fact, a significant negative correlation between free vaginal glycogen and progesterone level was recently reported in genital fluid samples of premenopausal women (42).

Also, the estrogenization and the subsequent production of glycogen by the vaginal epithelium have been shown to promote infection by *Candida albicans* (69). Glycogen was suggested to be a suitable substrate for *C. albicans*. This was particularly seen in post-menopausal women on systemic or vaginal estrogen therapy, whereas in a more recent study with premenopausal women, vulvovaginal candidiasis was observed in women with low vaginal α-amylase activity (43). There is a need to investigate the relationship between α-amylase activity and *Lactobacillus* dominance in post-menopausal women on and without estrogen therapy.

## Pathogenesis of stress-induced vaginal dysbiosis

The effect of stress on the incidence of BV may be mediated by stress-related dysregulation of immune function rather than behavioral changes associated with stress (20). An optimal immune response is required to prevent proliferation of BV-associated anaerobes. A sub-optimal response perhaps secondary to genetic polymorphism increases the risk of infection (70), and may allow more ascending genital tract infection (71). Stress enhances the progression of infection (including BV) and its pathophysiologic consequences (72, 73).

Perceived psychosocial stress is significantly and independently related to incidence and prevalence of BV in pregnant (74) and non-pregnant women (20). Some stress stimuli that can increase the risk of BV independent of individual behaviors include challenging life conditions such low income, poor housing, and dangerous neighborhood conditions, poor nutrition and interpersonal conflicts (73, 75). These studies assessed the level of stress exposure using the Cohen Perceived Stress Scale (20, 74, 75). Chronic psychosocial stress is also an established risk factor for preterm delivery independent of other biomedical and behavioral risk factors (2). Stress regardless of pregnancy status can range from acute and severe (e.g., trauma), moderate (e.g., change in life events), and chronic (e.g., standing for long hours as part of daily occupation). Nevertheless, during pregnancy, both short- and long-term stress exposure can lead to adverse pregnancy and delivery outcome (5, 76–78), though some studies with *in vitro* fertilization (IVF) patients argue otherwise showing only minimal association between prenatal stress and adverse pregnancy outcome (79).

Stress-induced cortisol binds to glucocorticoid receptors expressed on a range of immune cells and alters NF-κB activities, which regulates the activity of inflammatory mediators such as cytokines (IL-1β, IL-6, TNF-α, IFNγ) and chemokines (IL-8, CCL5). Glucocorticoids also facilitates immunosuppression by inhibiting proliferation, migration and cytotoxicity of lymphocytes and leukocytes, and secretion of IL-2 and IFN-γ (73). On the other hand, stress-induced epinephrine and norepinephrine bind to adrenergic receptors, activate cAMP and stimulate the transcription of genes encoding for a variety of inflammatory mediators (9, 80). Higher stress scores correlate with higher levels of IL-6 and TNF-α; and with low levels of the anti-inflammatory cytokine IL-10 (5). Furthermore, stress hormones increase vulnerability to infections that are primarily prevented by innate and adaptive immune responses by differentially regulating monocyte, macrophage, Th1/Th2 cells and cytokine expression patterns (81–84). These cortisol and catecholamines-mediated changes in gene expression dysregulate immune function (9). However, with previous observation of stress-related alterations in production of inflammatory mediators that are not associated with serum cortisol levels (85), the impact of stress on immune function may not be limited to cortisol-mediated effects alone (20).

**Figure 1:**
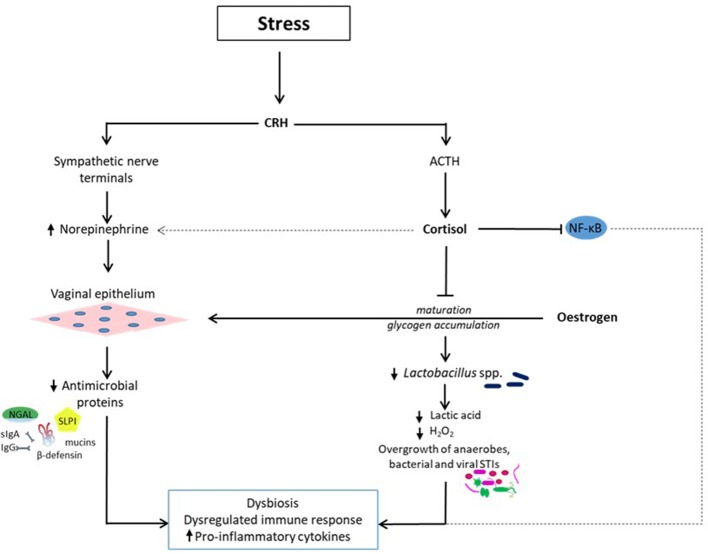
Stress-related reduction in vaginal *Lactobacillus* dominance (dysbiosis) and dysregulated immune response. Exposure to psychosocial stress induces the release of cortisol and norepinephrine via the hypothalamic-pituitary-adrenal and SAM axes respectively. Cortisol inhibits the estrogen-related vaginal epithelial maturation and glycogen accumulation. Levels of vaginal free glycogen and *Lactobacilli* are reduced leading to decreased lactic acid and hydrogen peroxide (H_2_O_2_) synthesis and pH. Consequently, a dysbiotic environment conducive for the proliferation of bacterial vaginosis-associated anaerobic bacteria such as *Gardnerella, Prevotella, Mobiluncus, Atopobium, Megasphera*, and sexually transmitted infections such *Neisseria gonorrhea, Chlamydia trachomatis*, human immunodeficiency virus is created. Cortisol also affects immune response by altering the nuclear factor-κB (NF-κB) signal transduction pathway, which regulates inflammatory gene expression. These effects are exacerbated by the concurrent release of norepinephrine, which binds to vaginal epithelial cells and potentiates the pro-inflammatory response via a reduction in the release of antimicrobial proteins including mucins, immunoglobulins (secretory Ig A and IgG), β-defensins, secretory leucocyte protease inhibitor (SLPI), and neutrophil gelatinase-associated lipocalin (NGAL). The overall effect is a dysbiotic vaginal ecosystem with a sub-optimal immune response, which encourages upper genital tract infection with deleterious gynecological and obstetric sequelae.

A stress-induced vaginal dysbiosis with disrupted vaginal mucosal and immune response-related proteins (e.g., lactoferrin), reduction in neutrophil bactericidal potency and reduced abundance of commensal *Lactobacillus* have been demonstrated in a mouse model (86). Reduced *Lactobacillus* was associated with overgrowth of other microbes (86), most likely anaerobic and facultative bacteria. Exposure to stress produces a significant decrease in the abundance of vaginal *Lactobacillus* species and can amplify the severity and consequences of vaginal infection (27). This occurs through the release of CRH, which stimulates sympathetic nerve terminals and the SAM axis to release norepinephrine. Norepinephrine can enter the vagina from the bloodstream and is locally secreted by vaginal epithelial cells to which it binds (80). Though the vaginal epithelial cells in this *in vitro* experiment were exposed to a high range of norepinephrine concentrations (1-10 μM), this may not differ significantly from the *in vivo* environment. This is supported by evidence of abundant supply of norepinephrine from nerve terminals innervating the cervicovaginal mucosa in humans and animals (87–91) and vaginal epithelial cells could potentially interact with norepinephrine in the circulation as well (80). In addition, local production of norepinephrine by epithelial cells in other sites such as the cornea has been demonstrated (92). The combined effect of a depleted vaginal *Lactobacillus* dominance, which leads to decreased lactic acid and H_2_O_2_ production and increased alkalinity and proliferation of pathogenic bacteria; and increased norepinephrine secretion result in a heightened pro-inflammatory response with increased production of cytokines and chemokines [(27); Figure 1). In essence, high norepinephrine levels induced by severe stress, potentiates the pro-inflammatory response of vaginal epithelial cells possibly in an autocrine fashion in the presence of diminished *Lactobacilli* and low pH (80).

It also noteworthy that the incidence and prevalence of BV is influenced by other factors including frequent unprotected sexual activity with new or multiple partners (93–96), smoking, alcohol and drug use (73, 96–99), contraceptive practice (97, 98, 100), vaginal douching (95, 97, 101), menstruation (102, 103), pregnancy (96, 104), low educational and socioeconomic status, and black race (73, 95–98, 100, 105). Nevertheless, after adjusting for all of these associated factors that usually account for only minimal proportion of the variation in BV, the relationship between psychosocial stress and aberrant vaginal microbiota has been shown to be maintained in several studies (20, 74). The pathophysiology, risk factors and consequences of BV and other female genital tract infections have been reviewed extensively (22, 106), and more investigation is recommended due to the enigmatic nature of these conditions especially BV.

## Cortisol inhibits vaginal glycogen deposition

The vaginal glycogen deposition action of estrogen was demonstrated over 50 years ago. Wrenn and colleagues in an attempt to determine the estrogen content of biological fluids, observed a rapid increase in vaginal glycogen content after locally administering estrogen extracted from human and cow urine and blood samples to adolescent, ovariectomized rats (58). This was termed the “vaginal glycogen assay for estrogen.” Furthermore, they investigated the specificity of the assay by comparing the effect of 17β-oestradiol with progesterone, cortisol, deoxycorticosterone, testosterone and diethylstilboestrol (DES, synthetic non-steroidal estrogen), both individually and in combination with 17β-oestradiol. Of all these hormonal substances, which are equally applied intravaginally, only DES stimulated a vaginal glycogen response singly, which was significantly enhanced in combination with 17β-oestradiol. Interestingly, the hormones associated with stress i.e., cortisol (30 μg/0.01 ml) and deoxycorticosterone (40 μg/0.01 ml) exhibited moderate inhibition of the glycogen response when administered in combination with 17β-oestradiol, while progesterone and testosterone were ineffective in this regard. In addition, the vaginal glycogen action of the other two naturally occurring forms of estrogen was tested. It was observed that oestrone which is usually found in post-menopausal women was only about 10% as active as 17β-oestradiol, whereas oestriol (common during pregnancy) was as potent as 17β-oestradiol (58). This gives more credence to the more stable *Lactobacilli* dominated vaginal microbiota rich in glycogen and lactic acid content observed in healthy pregnant women. However, because this experiments were conducted in rats with relatively high concentrations of cortisol (8.3 × 10^6^ nmol/l) compared to circulating cortisol levels under maximum stress such as major surgery (726 and 1297 nmol/l) (107, 108) or intravenous administration of 50 mg hydrocortisone (2450 nmol/l) (109) in humans, more empirical evidence from human experiments is required. Perhaps the local (cervicovaginal) concentrations of cortisol as demonstrated by Wrenn et al. (58) is more crucial in the glycogen response than the influence of circulating cortisol as reported in humans. More so, it could be that such repressive effect on vaginal glycogen deposition in humans could only be attained via a combined local action of cortisol and norepinephrine that mediate the physiologic stress response. This is yet to be demonstrated in human specimens.

The above evidence could be one mechanism through which frequent use of corticosteroids alters the equilibrium of the vaginal microbiome apart from dysregulated immune response. Cortisol repressed the estrogen-related maturation of vaginal epithelial cells and glycogen accumulation. If this happens in humans, the breakdown of glycogen to smaller polymers by α-amylase is reduced leading to low lactic acid production and ultimately loss of *Lactobacillus* dominance. This recipe creates a conducive, less acidic ecosystem for infection by strict and facultative anaerobic bacteria, viruses, fungi, and protozoa (27). Because the maturation of the vaginal epithelium is impaired, there is inadequate secretion of mucins and other antimicrobial proteins such as secretory leukocyte protease inhibitor (SLPI), neutrophil gelatinase-associated lipocalin (NGAL), and β-defensins. In addition, cortisol also alters NF-κB activity and dysregulates the expression of pro-inflammatory cytokines and chemokines (9). The combined effect is a dysregulated/ineffective inflammatory response against the pathogens, and an uncontrolled and possibly ascending genital tract infection, which can be deleterious especially during pregnancy.

Since stress is almost inevitable, these actions of cortisol have a propensity to disrupt the equilibrium of the vaginal microbiome and its capacity to combat infectious agents. Excessive exposure to psychosocial stress is independently associated with increased prevalence of BV (20, 73–75), which is the most common vaginal condition in women of reproductive age. BV is a quintessential dysbiotic condition characterized by overgrowth of anaerobic gram-negative and gram-variable bacteria secondary to a decrease in the protective *Lactobacillus* species. During pregnancy, BV has been linked to stress and it increases the risk of preterm labor by ~3-fold (110). Infections such as BV can lead to ascending intrauterine infection that stimulates immune responses with release of inflammatory mediators, uterotonins (Prostaglandins, PGs) and extracellular matrix degrading enzymes (e.g., matrix metalloproteinases, MMPs). These inflammatory cytokines, chemokines, PGs, and MMPs stimulate the pathway to premature rupture of membranes and preterm birth i.e., uterine contraction, cervical ripening, and membrane activation, via a positive feedback loop (22, 28, 106, 110).

While studies on stress and vaginal dysbiosis are often done without measurement of cortisol levels (20, 74, 75, 111); and others did not find a clear pattern of association between perceived stress (resulting from change in relationships, sickness/injury, finances, work pressure or routines, unpleasant events, and relationship pressure) (111) or stress hormones and BV (112); the disruptive role of cortisol on the vaginal microbiota and immunity has been highlighted by the experiments of Wrenn et al. (58), albeit in animal models studied with significantly higher cortisol concentrations. Also, the correlation of cortisol levels with the prevalence of BV and other genitourinary tract infections in humans has been reported (113). In fact, a significant increase in cortisol across the 2nd and 3rd trimesters was observed in patients with BV, an association hypothesized to be restricted to a local response to cortisol (113). Elevated cortisol levels could correlate with reduced vaginal glycogen content, loss of *Lactobacillus* dominance, decreased acidity, dysbiosis, and increase production of pro-inflammatory mediators. More so, it is plausible that increased norepinephrine activity, which in concurrence with cortisol is involved in the “fight-or-flight” response and has been shown to potentiate the pro-inflammatory response of vaginal epithelial cells, amplifies these actions of cortisol (Figure 1). Furthermore, since vaginal glycogen deposition is not required for immediate survival during exposure to stressful stimuli and subsequent “fight-or-flight” response, cortisol's action appears to be an adaptive mechanism to preserve energy sources for organs such as the brain, cardiac and skeletal muscles albeit with unpleasant reproductive consequences when prolonged. A more comprehensive investigation to elucidate the mechanisms of cortisol's action on the human vaginal microflora and the consequent gynecological and obstetric health implications is required.

## Stress-induced altered maternal vaginal and offspring gut microbiota

Exposure to chronic psychosocial stress during pregnancy may amplify the physiologic pregnancy-induced immunosuppression (114). This can increase the risk of dysbiosis, recurrent genitourinary infection and eventual loss of the beneficial lactobacillus-dominated vaginal microbiota as seen in BV and candidiasis (115, 116). Since the initial neonatal gut microbial colonization is dependent on vertical transmission from the maternal vaginal microbiota during parturition, acquisition of a dysbiotic lactobacillus-depleted microbiota predisposes the infant to altered gastrointestinal tract maturation, impaired extraction of energy and macromolecules essential for normal growth and development, and dysfunctional immune system. This eventually increases the infant's risk of disturbed energy metabolism, obesity, insulin resistance, and diabetes mellitus. Other conditions include diarrheal illness, food allergy, atopic diseases, and inflammatory bowel disease, and irritable bowel syndrome (117, 118). Also, the risk of long-term neurodevelopmental disorders due to reprogramming of the developing brain has been reported (86, 114, 119). These findings are supported by human studies and experiments with animal models. For instance, infants of mothers with high prenatal stress (reported and due to high cortisol levels) had significantly lower relative abundance of lactic acid-producing bacteria including *Lactobacillus, Lactoccus, Aerococcus*, and *Bifidobacteria*; and higher relative abundance of Proteobacteria including the pathogenic *E. coli, Serratia*, and *Enterobacter*. This dysbiotic microbiota is potentially associated with an increased level of lipopolysaccharide (LPS)-induced inflammation and more maternally-reported infant gastrointestinal symptoms and allergic reactions (118).

Furthermore, female rhesus monkeys exposed to moderate stress (acoustic startle) for 6 weeks in later gestation showed activated HPA axis and increased cortisol levels (above those found in normal pregnancy), that was sufficient to cause altered intestinal microbiota characterized by decreased protective *Lactobacilli* and *Bifidobacteria* in their offsprings (120). Reduced concentrations of *Lactobacilli* and total and Gram-negative aerobes and facultative anaerobes have also been observed in the small intestine of pups whose mothers were injected with cortisone before birth (121, 122). These animal studies have provided ample evidence that the relationship between prenatal psychosocial stress and offspring programming is mediated, at least in part, by elevated cortisol levels (119). The pathophysiology of the effect of prenatal stress on maternal gut/vaginal and fetal gut microbial composition, and the development of insulin resistance, increased adiposity and metabolic syndrome later in life in humans is beyond the scope of this review but requires further investigation.

## Preventive and treatment strategies for stress-induced vaginal infection and inflammation

Optimal preventive/treatment approach to counteract the effect of stress on vthe aginal microenvironment should aim to alleviate or eliminate the stress stimuli and the associated infectious and inflammation agent(s). Though stress is almost inevitable, some life modifications can help an individual cope with stressors better. These adaptive measures include regular physical exercise, healthy eating, having adequate sleep and avoiding unhealthy habits like smoking, alcohol, and drug abuse. In addition to eliminating stressors and lifestyle modifications, appropriately administered antibiotics or antifungal agents can eliminate bacterial and fungal infections while inflammation can be targeted pharmacologically using anti-inflammatory agents. This is an important therapeutic goal because inflammation can persist even after the stress stimuli or infectious agents have been eliminated (22). Antibiotic treatment may also alter the vaginal bacterial composition with potential colonization by opportunistic pathogenic agents (123).

Another potential approach to restoring and/or maintaining vaginal homeostasis that avoids the adverse effect of antibiotics on beneficial microbiota (124) is the use of probiotics e.g., *Lactobacillus* species (*L. crispatus*), and prebiotics such as D-lactic acid, glycogen, oligosaccharide etc. that stimulate probiotic bacterial growth (22, 124–131). Recently, in a study including 6 women, 5 of whom were pregnant, vaginal, and oral lactoferrin administration improved vaginal microbiota *Lactobacillus* dominance and prevented refractory BV, cervical inflammation, and preterm delivery (132). This has previously been attempted with similar results by the same group (133) and others (134); and gives credence to the observed altered vaginal microbiota (with decreased *Lactobacilli* and lactoferrin levels) when mice were exposed to mild prenatal stress (86). A combination of probiotics and prebiotics could provide clinically useful adjunct to pharmacological and other strategies for treating/preventing vaginal infections and women's health in general (129, 131).

Some anti-inflammatory agents that can block LPS-driven production of cytokines (e.g., TNF-α, IL-6 and IL-1β) as observed in stress-induced vaginal colonization by anaerobic bacteria include phosphodiesterase (PDE) inhibitors, MAP kinase inhibitors, TNF biologics and NF-κB inhibitors (135). Some of these agents have also been applied in the prevention of LPS-stimulated preterm birth and fetal death (136, 137). With further exploration of the clinical utility, safety and toxicity of these anti-inflammatory agents, various stages of the stress/infection-associated inflammatory process can be targeted. However, because NF-κB is the “master regulator” of inflammatory gene expression, there is the crucial concern that inhibiting the activation of NF-κB and/or other pro-inflammatory mediators could prevent optimal activation of host innate and adaptive immune responses and increase the risk of infection. This could be deleterious particularly in an immunocompromised state such as pregnancy (135, 138).

## Main findings and future direction

The potential action of the stress hormone cortisol in the maintenance of vaginal health has been highlighted. Stress stimuli are ubiquitous and women do not enjoy any exemptions. The physiologic “fight-or-flight” response may be deleterious to their lower genital tract microbiome if the stress stimuli persist for longer than necessary. Persistent exposure to psychosocial stress and stimulation of the HPA and SAM axes, with a corresponding increase in cortisol and norepinephrine levels are associated with dysbiosis and increased susceptibility to several infections including genitourinary tract infections. Though this could be solely due to a dysregulated immune response, a cortisol-induced inhibition of vaginal glycogen deposition may be involved especially in the instance of vaginal infection. The estrogen-related increased vaginal glycogen deposition and epithelial maturation are requirements for the maintenance of vaginal eubiosis. The ability of cortisol to disrupt this process as shown in animal models is important in the pathogenesis of vaginal dysbiosis and subsequent development of infection and inflammation. If proven in humans, this phenomenon may be more crucial in pregnancy where a healthy Lactobacillus-dominated vaginal microbiome is sacrosanct, and there is local production of more CRH from gestational tissues including the decidua, fetal membranes (chorioamnion), and placenta. Because the current evidence of the repressive role of cortisol on vaginal glycogen deposition involve experiments in animals with high concentrations of cortisol compared to that observed during severe stress in humans, further work especially in humans is needed to elucidate the pathophysiologic association between cortisol levels and vaginal microbiomial architecture and function.

## Author contributions

EA conceived the research idea and conducted the literature search. EA and DA contributed to writing the manuscript. Both authors revised and approved the submission of the final manuscript.

### Conflict of interest statement

The authors declare that the research was conducted in the absence of any commercial or financial relationships that could be construed as a potential conflict of interest.

## Abbreviations

ACTH: adrenocorticotropic hormone
BV: bacterial vaginosis
cAMP: cyclic adenosine monophosphate
CCL5: Chemokine (C-C motif) ligand 5
CRH: corticotropin-releasing hormone
DES: diethylstilboestrol
HPA: hypothalamic-pituitary-adrenal
IFNγ: interferon gamma
IL: interleukin
IL-1RA: interleukin-1 receptor antagonist
NF-κB: nuclear factor kappa-light-chain-enhancer of activated B cells
NGAL: neutrophil gelatinase-associated lipocalin
PVN: paraventricular nucleus
SAM: sympathetic-adrenal-medullary
SLPI: secretory leucocyte protease inhibitor
TLR: toll-like receptors
TNF-α: tumor necrosis factor alpha.
